# Sex Differences in Peripheral Mu-Opioid Receptor Mediated Analgesia in Rat Orofacial Persistent Pain Model

**DOI:** 10.1371/journal.pone.0122924

**Published:** 2015-03-25

**Authors:** Xiaofeng Bai, Xia Zhang, Yanshu Li, Li Lu, Bo Li, Xiaofan He

**Affiliations:** 1 Associate Professor, Department of Oral and Maxillofacial Surgery, School & Hospital of Stomatology, China Medical University, 117 North Nanjing Street, Shenyang, P. R. of China, 110002; 2 Associate Professor, Department of Anesthesiology, School & Hospital of Stomatology, China Medical University, 117 North Nanjing Street, Shenyang, P. R. of China, 110002; 3 Assistant Professor, Department of Cell Biology, China Medical University, 92 Bei'er Road, Shenyang, P. R. of China, 110001; 4 Professor, Department of Oral and Maxillofacial Surgery, School & Hospital of Stomatology, China Medical University, 117 North Nanjing Street, Shenyang, P. R. of China, 110002; 5 Associate Professor, Department of Oral Anatomy and Physiology, School of Stomatology, China Medical University, 117 North Nanjing Street, Shenyang, P. R. of China, 110002; 6 Assistant Professor, Department of Anesthesiology, School & Hospital of Stomatology, China Medical University, 117 North Nanjing Street, Shenyang, P. R. of China, 110002; Xi'an Jiaotong University School of Medicine, CHINA

## Abstract

Unilateral ligation of the tendon of anterior superficial part of rat masseter muscle (TASM) leads to long-lasting allodynia. Sex differences in peripheral mu-opioid receptor (MOR)-mediated analgesia under persistent myogenic pain are not well understood. In this study, we examined (1) whether locally applied MOR agonists attenuate persistent pain following TASM ligation in a sex dependent manner, (2) whether there are sex differences of MOR expression changes in rat trigeminal ganglia (TG). The effects of MOR agonist, D-Ala2, N–Me-Phe4, Gly5-ol]-Enkephalin acetate salt (DAMGO), were assessed 14 days after TASM ligation in male, female and orchidectomized (GDX) male rats. MOR mRNA and protein levels in TG 14 days following tendon ligation were also determined. The mechanical thresholds of the injured side were significantly decreased in both male and female rats, from 3 days to 28 days after TASM ligation. A10 μg DAMGO significantly attenuated allodynia in male rats. A 10-fold higher dose of DAMGO was required in female and GDX male rats to produce the level of anti- allodynia achieved in male rats. The level of MOR mRNA in TG from male rats was significantly greater 14 days after TASM ligation compared with the sham-operated male rats, but not from female and GDX male rats. After TASM ligation, males had significantly more MOR immunoreactivity in TG compared to sham-operated males. The MOR levels increased to 181.8% of the sham level in male rats receiving tendon injury. But there was no significant change in female rats receiving tendon injury compared to the sham female rats. Taken together, our data suggest that there were sex differences in the effects of peripheral MOR agonists between male and female rats under TASM ligation developing long-lasting pain condition, which is partly mediated by sex differences in the changes of MOR expressions and testosterone is an important factor in the regulation of MOR.

## Background

The functional role of peripheral mu-opioid receptor (MOR) in producing anti-nociception as well as anti-hyperalgesia has been demonstrated in various pain models [1,2], and is partly mediated by the significant increase in the MOR mRNA and protein levels in dorsal root ganglia [3]. For example, local administration of MOR agonists attenuates hyperalgesia associated with neuropathic pain [4] and inflammatory pain [5,6].

Disorders of the temporomandibular joint (TMJ) and muscles of mastication often lead to chronic pain conditions which are difficult to treat and become a major health problem. The currently used orofacial inflammatory models generate pain lasting from several hours to 2 weeks; hence they are not suitable for studying the long persistency of chronic myogenic pain in TMJ disorders. However, unilateral ligation of the tendon of anterior superficial part of rat masseter muscle (TASM) leads to long-lasting and constant mechanical allodynia [7,8], which is particularly useful in studying the chronicity of myogenic pain.

Analgesia to systemic opioid treatment shows sex differences in human and animals [9,10,11]. At the peripheral site, activation of MOR produces more potent analgesia in male than in female rats in inflammatory and visceral pain model [6,12]. However, additional studies with different pain models are required for our overall understanding on neurobiological mechanisms underlying sex different responses to local MOR agonist treatment. Thus, it would be interesting to know whether there are sex differences in peripheral MOR-mediated analgesia under persistent myogenic orofacial pain. In this study, we investigate (1) whether locally applied MOR agonists attenuate persistent pain following ligation of the tendon of masseter muscle in a sex dependent manner, (2) whether there are sex differences of MOR expression changes in rat trigeminal ganglia (TG) in a TASM ligation-induced long-lasting pain condition, (3) whether testosterone involves in analgesia in male rats.

## Methods

### Animals

Adult male, female and orchidectomized (GDX) male Sprague–Dawley rats (8 weeks, 200–250 g) from the Experimental Animal Center of China Medical University were used in the present study. Male rats (5 weeks) were performed gonadectomy surgery. GDX rats were used three weeks after gonadectomy surgery. All animals were housed in a temperature-controlled room under a 12:12 light-dark cycle for at least 1 week to acclimate to the surroundings and with free access to food and water. The study was approved by the Ethical Committee of Animal Research at the China Medical University. For behavioral experiments, 6 rats were used per group. Experimental and Sham groups for real-time PCR and Western blot studies consisted of 6 rats per group. The ligation procedure disrupted estrous cycle, therefore estrous cycle in female rats was not determined in this study.

### Persistent myogenic orofacial pain model

TASM was achieved via an intraoral approach according to Guo et al [7]. Animals were anesthetized with pentobarbital sodium (50 mg/kg, intraperitoneal injection). On the left intraoral site, a 3-mm-long incision was made posterior-anteriorly lateral to the gingivobuccal margin in the buccal mucosa, beginning immediately next to the first molar. The TASM was gently freed from surrounding connective tissues and tied with two chromic gut (4.0) ligatures, 2-mm apart. The sham-operated rats received the same procedure except tendon ligation.

### Behavioral studies

All behavioral tests were conducted under blind conditions by an investigator who did not know the treatments as previously described [13]. A series of calibrated von Frey filaments with bending forces ranging from 0.19 g to 118 g were applied to the skin above the TASM. An active withdrawal of the head from the probing filament was defined as a response. Each von Frey filament was applied 5 times at intervals of a few minutes. The response frequencies [(number of responses/number of stimuli) X 100%] to a range of von Frey filament forces were determined and a stimulus-response frequency curve (S-R curve) was plotted. After a non-linear regression analysis, an EF50 value, defined as the von Frey filament force (g) that produces a 50% response frequency, was derived from the S-R curve. The EF50 value was used as a measure of mechanical sensitivity. A decreased EF50 suggested the presence of mechanical allodynia.

Mechanical threshold was determined before and 3 days, 7 days, 14 days and 28 days after tendon ligation. D-Ala_2_, N–Me-Phe4, Gly5-ol]-Enkephalin acetate salt (DAMGO) is a mu-opioid receptor agonist. The effect of DAMGO (Tocris, MS, USA) on mechanical threshold was examined on the 14^th^ day post tendon ligation, a time point at which mechanical allodynia was pronounced. Four different doses of DAMGO were prepared to a final concentration of 1μg/50 μl, 10 μg/50 μl, 50 μg/50 μl, and 100 μg/50 μl in phosphate buffered saline (PBS) and injected into TASM 14 days after tendon ligation. Four different doses of DAMGO were prepared by an investigator. All drug injections were made via a 27 gauge needle under inhalation anesthesia by another investigator who did not know the dose of DAMGO. Injections were made for 5–10 seconds. The rats in Control groups received PBS injection in the same manner.

The effect of opioid receptor antagonist, naloxone methiodide from Sigma (St. Louis, MO) on mechanical threshold was examined on the 14^th^ day post tendon ligation. Naloxone methiodide (1 mg/kg) or PBS was injected to TASM 10 minutes before 100 μg DAMGO injection in male and female rats and the results were collectively plotted.

### Real-time PCR

All animals were killed with a lethal dose of sodium pentobarbital. Total RNAs were extracted from left TG of male, female and GDX male sham-operated rats or rats 14 days following tendon ligation without drug injection. All RNAs were isolated from TG using Trizol (Invitrogen, Carlsbad, CA, USA) and purified according to the RNeasy kit (Qiagen, MD, USA) that included a DNase treatment to remove genomic DNA. RNA extracts were quantified by the absorbance at 260 nm (A_260_). The purity of extracts was assessed by the ratio of A_260_ vs. A_280_ and only those having the ratio above 1.9 were used for PCR. SuperScript II (Invitrogen) was used to generate cDNA from 500 ng of RNA along with 2.5 ng of random primer per reaction. Real-time PCR analysis of cDNA was then performed using Maxima SYBR Green/ROX qPCR Master Mix in an Eppendorf Mastercycler Ep Realplex 2.0 (Fermentas, Forest City, CA, USA). Primers specific to MOR cDNA were 5′-GCC CTC TAC TCT ATC GTG TGT GTA -3′(forward) and 5′-GTT CCC ATC AGG TAG TTG ACA CTC-3′ (reverse). Primers specific to actin cDNA were 5′-GGT CCA CAC CCG CCA CCA G-3′ (forward) and 5′-CAG GTC CAG ACG CAG GAT GG-3′ (reverse). We obtained the ratios between MOR and actin to calculate the relative abundance of mRNA levels in each sample. Relative amount of the MOR mRNA was calculated by the comparative CT method (ΔΔCT method) between sham and experimental groups [14].

### Immunohistochemistry

The male and female sham-operated rats or rats 14 days following tendon ligation were sacrificed. Rats were perfused transcardially with by 4% paraformaldehyde in PBS (250 ml; pH 7.2). The left TG from each rat was extracted and sectioned coronally at 12 μm. The conventional procedures of immunohistochemistry were performed with antibodies against MOR (24216, ImmunoStar, 1:250). For immunofluorescence the sections were incubated at 37°C for 30 minutes with Cy-3 conjugated goat anti-rabbit antiserum (JacksonImmuno, 1:250).

### Western Blot

Total proteins were extracted from left TG of male and female sham-operated rats or rats 14 days following tendon ligation. The tissues were homogenized in RIPA buffer containing protease inhibitor cocktail. The protein concentration was determined using Bio-Rad protein assay kit (Bio-Rad, USA). Each sample contains 50 micrograms of protein. The membrane was blocked with 5% milk Tris buffered saline in one hour at room temperature then incubated with primary antibodies for MOR (1:1000, Millipore AB5511, USA) [6]. Bands were visualized using ECL (Western Lightning, USA). Protein level for MOR was normalized to that of β-actin in the same sample. Data from tendon ligation rats were normalized to that of sham rats.

### Data analysis

The time-dependent changes in mechanical thresholds (EF50) before and after drug treatments were analyzed with a two-way analysis of variance (ANOVA) with repeated measures. MOR mRNA data were analyzed with a one-way ANOVA on means or Kruskal-Wallis one-way ANOVA on ranks depending on the outcome of a normality test. All multiple group comparisons were followed by Dunnett’s post hoc test. Data were presented as mean ± SEM and *p* < 0.05 was considered significant.

## Results

### Persistent behavioral allodynia following tendon ligation

**Fig 1:**
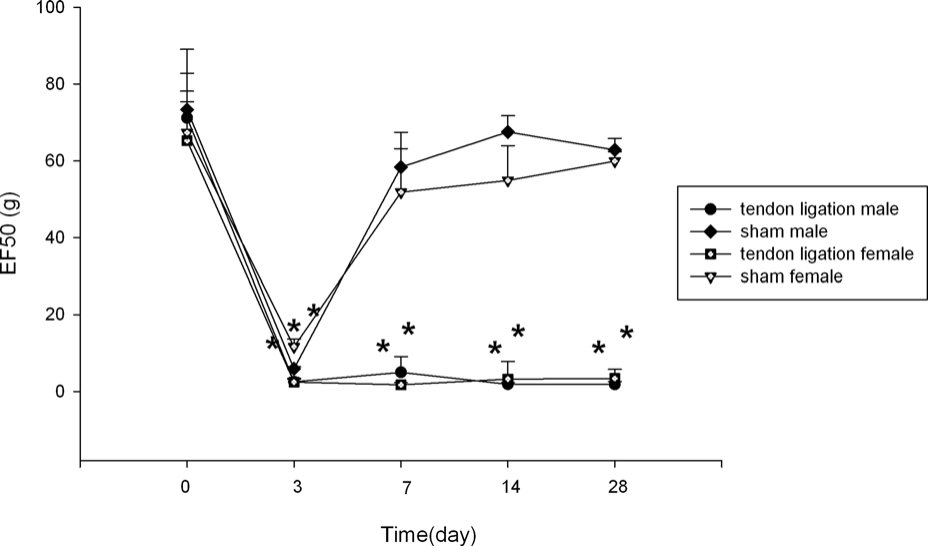
Mechanical allodynia following ligation of the masseter tendon. The EF50 were derived from the respective stimulus-response frequency function curves. A significant reduction of EF50 suggested the presence of mechanical allodynia. All data are shown as mean ± SE and each group consisted of 6 animals. * denotes significant effects with respect to the baseline at *p* < 0.05.

After TASM ligation, the mechanical thresholds (EF50) of the injured side were significantly decreased (Fig 1) in both male and female rats, indicating that hyperalgesia/allodynia had been successfully developed. The reduction of EF50 was statistically significant on 3 days post ligation (*p* < 0.001) and the allodynia persisted throughout the observation up to 28 days post ligation (*p* < 0.001) in male and female rats. There were no significant sex differences of EF50 at all time points.

In male and female sham-operated rats, the EF50 on the injured side was reduced on 3 days post ligation compared to the baseline levels (*p* < 0.001). And the mechanical thresholds recovered after 7 days post ligation.

### Sex differences in anti—allodynia at the peripheral MOR

**Fig 2:**
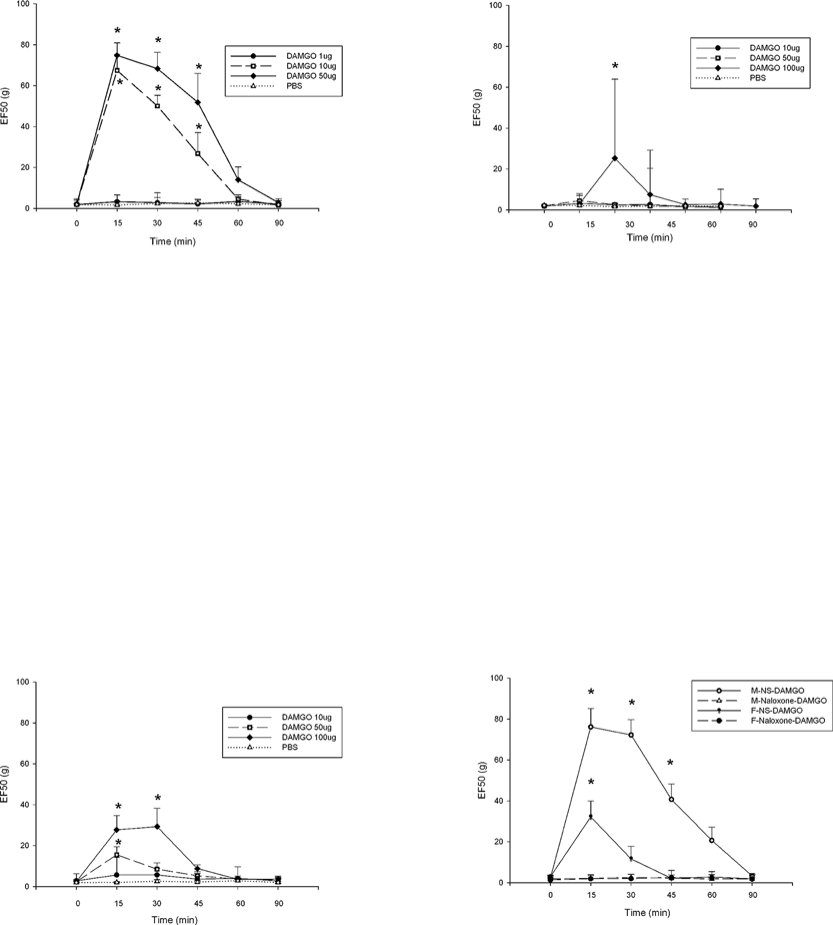
The sex differences effects of opioid receptor agonist and antagonist on mechanical sensitivity. Effects of injection in the masseter tendon with DAMGO or PBS on mechanical sensitivity 14 days after tendon ligation in male (2A), female (2B) and GDX male rats (2C). The effects of opioid receptor antagonist were showed in Fig 2D. All data are shown as mean ± SE and each group consisted of 6 animals. * denotes significant effects with respect to the pre-treatment condition at *p* < 0.05.

There were no effects when PBS had been injected into the injured masseter tendon in male, female and GDX male rats. Treatment of the masseter tendon with DAMGO dose-dependently reversed the allodynia in male rats (Fig 2A). There was a significant treatment effect (F = 107.7, *p* < 0.001) and time effect (F = 44.3, *p* < 0.001). A significant interaction between dose and time was observed (F = 15.3, *p* < 0.001). DAMGO at 1 μg did not attenuate the allodynia in male rats (F = 44.3, *p* < 0.001). DAMGO at 10 μg and 50 μg blocked mechanical allodynia in male rats. In female rats, there was a significant treatment effect (F = 8.3, *p* = 0.002) and time effect (F = 15.6, *p* < 0.001). In GDX male rats, there was a significant treatment effect (F = 9.1, *p* = 0.002) and time effect (F = 15.3, *p* < 0.001). A significant dose and time interaction was also observed in female rats (F = 9.7, *p* < 0.001) and GDX male rats (F = 6.1, *p* < 0.001). A 10 fold higher dose of DAMGO (100 μg) partially, but significantly, attenuated the allodynia in female rats (Fig 2B) and GDX male rats (Fig 2C).

The 10 μg dose of DAMGO significantly attenuated the allodynia for 45 minutes in male rats (t = 6.088, *p* < 0.001). But the 10 μg dose of DAMGO did not change the mechanical thresholds significantly in female and GDX male rats. The 50 μg dose of DAMGO increased the mechanical thresholds in male rats for 45 minutes (t = 7.475, *p* < 0.001). The 50 μg dose of DAMGO increased the mechanical thresholds in GDX male rats only for 15 minutes (t = 3.978, *p* < 0.001). The 50 μg dose of DAMGO was still ineffective when given in female rats (t = 1.301, *p* > 0.05). DAMGO was ineffective when injected in the contralateral masseter suggesting that the drug effects are not due to systemic responses (data not shown).

The effects of opioid receptor antagonist were showed in Fig 2D. The mechanical thresholds in male rats with naloxone were significantly lower than those in rats with PBS 15 minutes, 30 minutes and 45 minutes after DAMGO administration (*p* < 0.001). In female rats, the mechanical thresholds with naloxone were significantly lower than those with PBS 15 minutes after DAMGO administration (*p* < 0.001).

### Sex differences in MOR expression in TG following tendon ligation

**Fig 3:**
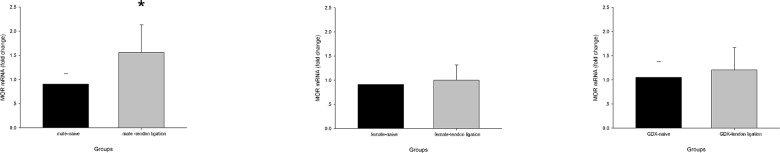
MOR mRNA levels in TG of male, female and GDX male rats. MOR mRNA levels in TG of age-matched male (3A), female (3B) and GDX male (3C) rats 14 days after tendon ligation. All data are shown as mean ± SE and each group consisted of 6 animals. * denotes significant effects with respect to sham-operated condition at *p* < 0.05.

In order to compare the impact of injury on MOR expression between the sexes, we measured the changes of the MOR mRNA content in TG 14 days after TASM ligation. In male rats, the level of MOR mRNA in TG was significantly greater following TASM ligation compared with the sham-operated male rats (Fig 3A). TASM ligation did not alter MOR expression in TG from female (Fig 3B) and GDX male rats (Fig 3C).

**Fig 4:**
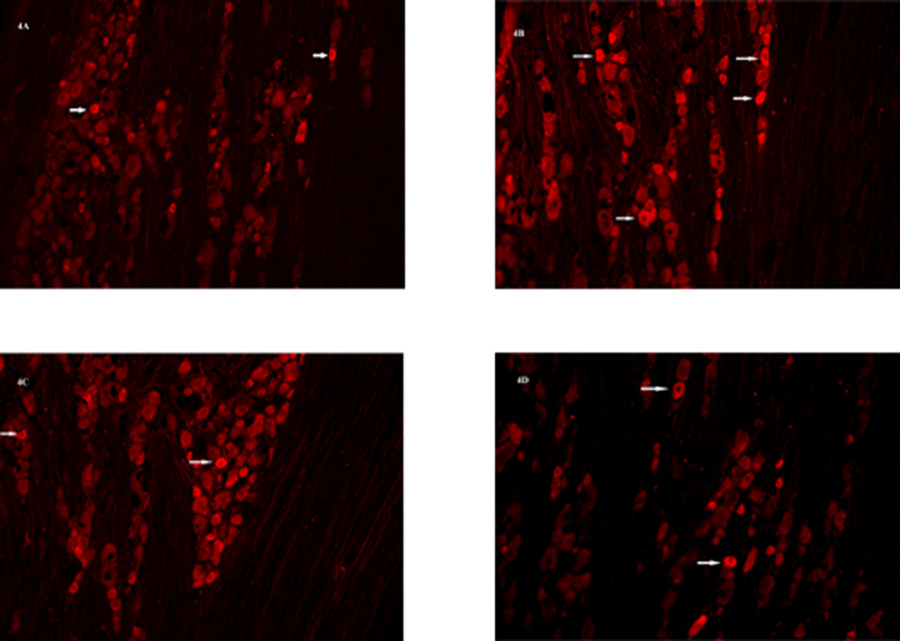
MOR positive cell levels in TG of male and female rats. MOR positive cell levels in TG of sham-operated male (4A), sham-operated female (4C), male (4B) and female rats (4D) 14 days after tendon ligation.

There were no significant differences of MOR positive cell levels in TG of sham-operated male (Fig 4A) and female rats (Fig 4C). Fourteen days after TASM ligation, males had significantly more MOR immunoreactivity in TG compared to sham-operated males (Fig 4B) (t = 3.477, *p* = 0.014). MOR positive cell levels did not change significantly in females after TASM ligation (Fig 4D).

**Fig 5:**
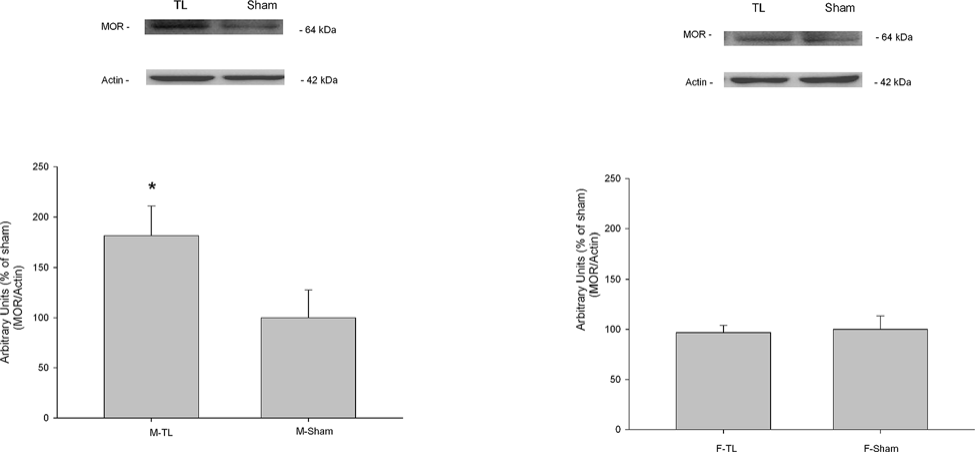
MOR protein level changes after tendon ligation in male and female TG. MOR protein levels in TG of male (Fig 5A) and female rats (Fig 5B) 14 days after tendon ligation. All data are shown as mean ± SE and each group consisted of 6 animals. * denotes significant effects with respect to sham-operated condition at *p* < 0.05.

The changes in MOR protein levels after tendon ligation in male and female TG were quantified by Western blot. The results indicated that the MOR levels increased to 181.8% of the sham level in male rats receiving tendon injury (t = 2.802, *p* = 0.049, Fig 5A). But there was no significant change in female rats receiving tendon injury compared to the sham female rats (t = 0.437, *p* = 0.685, Fig 5B).

## Discussion

The major finding of the present study is that a local administration of DAMGO can reverse the mechanical thresholds in long-lasting pain condition developed after TASM ligation. The mechanical thresholds with naloxone were significantly lower than those with PBS in male and female rats after DAMGO administration. There were sex differences in the changes of MOR expression and the effects of peripheral MOR agonist between male and female rats. Testosterone involves in analgesia in male rats.

Sex-related influences on pain have become an interesting topic, especially in the last 20 years. Clinically, the dose of both prescription and nonprescription analgesics is significantly higher among women than men [15,16]. In experimental pain models, several investigators have examined sex differences in analgesic responses. Most of them have focused on receptors in central nerve system [17,18]. To the best of our knowledge, there are no data regarding sex differences of anti-hyperalgasic effects of peripheral MORs in the orofacial tendon injury model, which is particularly useful in studying the chronicity of pain associated with TMJ disorders, sports injury, muscle overuse, and rheumatoid arthritis [7,8,19]. Our data showed that a 10 μg DAMGO significantly attenuated the allodynia in male rats. However, a 10 fold higher dose of DAMGO significantly attenuated the allodynia in female rats. This result is consistent with other studies in animals and human. Sex differences in anti-hyperalgesic effects of peripheral κ opioid receptor agonists become more evident at high doses under inflammatory pain conditions [20]. Moreover, a 10-fold higher dose of local δ-opioid receptor (DOR) agonist, [D-Pen², D-Pen^6^]-enkephalin (DPDPE) was required in female rats to produce the level of anti-hyperalgesia achieved in male rats [21]. Similarly, in human there are sex differences in the function of MORs. Female patients have higher pain indicators and require a larger amount of tramadol than male patients using intravenous patient-controlled analgesia [22]. Moreover, women experience more severe postoperative pain and require a greater dose of morphine than men in the immediate postoperative period [23].

Cellular mechanisms of sex differences in peripheral MOR-mediated analgesia have not been systematically investigated in long-lasting pain condition. In our study, there were significant differences between the levels of MOR mRNA and protein in male and female rat TG, which suggested sex differences in MOR-mediated responses result from the MOR expressions. MOR expressions are higher in male rats than those in female rats in many regions of central nerve system such as anterior pituitary, periaqueductal gray and midbrain [24,25,26]. In peripheral nerve system, sex differences in functional receptor expressions may also underlie sex specific analgesic effects. It is reported that sex differences in functional ATP-sensitive K^+^ channel (KATP) expression in TG may underlie sex specific responses to KATP agonists under an inflammatory pain condition [27]. Similarly, the cannabinoid receptor 1 (CB1R) and MOR expressions are higher in male rat TG than those in female rat TG under orofacial inflammatory condition [6,28].

This study demonstrated that a 10-fold higher dose of DAMGO was required in female and GDX male rats to produce the level of anti- allodynia achieved in male rats, suggesting a hormonal link between sex and analgesia. In GDX male rats, the effect of DAMGO was reduced due to low levels of testosterone. Recent clinical and experimental data provide evidences that testosterone contributions to pain. Women had a higher initial VAS score and required a greater dose of morphine than men in postoperative period. This sex-related difference disappeared in elderly patients [23], which suggests the effects of gonadal hormones on sex-related pain. Similarly, a study of transsexuals demonstrated that gonadal hormones affect the occurrence and incidence of pain. Eleven of the 47 male-to-female subjects undergoing estradiol/anti-androgen treatment develop chronic pain, whereas 6 of the 11 female-to-male subjects after testosterone administration report significant improvement of the chronic pain which is present before the beginning of hormone intake [29]. Studies of laboratory pain provide additional evidences that testosterone influences on pain responses. Testosterone, but not estradiol, is required for the regulation of CB1Rs in TG under inflammatory conditions, which provide explanations for the sex differences in the antihyperalgesic effects of peripherally administered cannabinoids [28]. Interestingly, the cytokine-induced up-regulation of MOR mRNA expression is prevented in TG from GDX male rats, which is restored with testosterone replacement. Testosterone plays a key role in the regulation of MOR in TG under the Freund's Complete Adjuvant (CFA)-induced inflammatory conditions [6]. However, it is reported that estrogen receptor signaling contributes to dynorphin and enkephalin regulation in mouse hippocampal mossy fiber pathway [30]. Additional studies with female rats of estrous stages or gonadectomized rats need to be conducted.

## Conclusions

Taken together, our data suggest that there were sex differences in the effects of peripheral MOR agonists between male and female rats under TASM ligation developing long-lasting pain condition, which is partly mediated by sex differences in the changes of MOR expressions and testosterone is an important factor in the regulation of MOR. The results from this study would offer important clues to the development of mechanisms regarding the sex differences in MOR function and sex-specific pharmacological treatment.
